# An interactive web-based programme on relapse management for people with multiple sclerosis (POWER@MS2) - development, feasibility, and pilot testing of a complex intervention

**DOI:** 10.3389/fneur.2022.914814

**Published:** 2022-09-23

**Authors:** Lisa Wenzel, Christoph Heesen, Julia Peper, Kristina Grentzenberg, Edeltraud Faßhauer, Jutta Scheiderbauer, Frithjof Thale, Björn Meyer, Sascha Köpke, Anne Christin Rahn

**Affiliations:** ^1^Medical Faculty and University Hospital Cologne, Institute of Nursing Science, University of Cologne, Cologne, Germany; ^2^Institute of Neuroimmunology and Multiple Sclerosis (INIMS), University Medical Center Hamburg-Eppendorf, Hamburg, Germany; ^3^Department of Neurology, University Medical Center Hamburg-Eppendorf, Hamburg, Germany; ^4^Nursing Research Unit, Institute of Social Medicine and Epidemiology, University of Lübeck, Lübeck, Germany; ^5^Deutsche Multiple Sklerose Gesellschaft, Bundesverband e.V., Hannover, Germany; ^6^MS-Stiftung Trier, Trier, Germany; ^7^GAIA AG, Hamburg, Germany

**Keywords:** multiple sclerosis, relapse management, decision aid, feasibility study, patient empowerment

## Abstract

**Introduction:**

Despite the lack of high-quality evidence regarding its long-term effectiveness, intravenous corticosteroid therapy is recommended as the standard treatment of acute multiple sclerosis relapses in Germany. High financial expenses and the equivalent effectiveness of oral corticosteroid therapy contrast with this trend. There is an urgent need to provide patients with evidence-based and comprehensible information on relapse management and to actively involve patients in relapse treatment decisions. Web-based decision support on relapse management could be an effective measure to empower people with multiple sclerosis making informed treatment decisions.

**Objectives:**

To develop a web-based programme on relapse management for people with multiple sclerosis and evaluate the feasibility and acceptability of the intervention.

**Methods:**

The study followed the first two phases of the UK Medical Research Council Framework for complex interventions. The first phase involved the development of an interactive web-based programme on relapse management. The second phase focused on the feasibility and pilot testing of the programme with people with multiple sclerosis and experts with a professional background in multiple sclerosis. Data was obtained using questionnaires with closed- and open-ended questions as well as qualitative semi-structured telephone interviews. Quantitative data was analyzed descriptively, whereas qualitative data was clustered by topic.

**Results:**

Feasibility of the intervention programme was tested with 10 people with multiple sclerosis and 10 experts. Feasibility testing indicated good practicability and acceptance of the content. After revision, the programme was piloted with seven people with multiple sclerosis and three experts. The results showed good acceptance in both groups. Based on the feedback, a final revision was performed.

**Conclusion:**

Feasibility and pilot testing indicated good user-friendliness, acceptance, and practicability of the programme. The programme is currently evaluated in a randomized controlled trial (Registration Number on ClinicalTrials.gov: NCT04233970). It is expected that the programme will have a positive impact on patients' relapse management and strengthen their autonomy and participation.

## Introduction

Multiple sclerosis (MS) is a neurological disorder that is substantially accompanied by uncertainties and impairment in quality of life for those affected (1). Even though progress has been made in recent years in terms of diagnosis and availability of treatment options, gaps remain in the epidemiologic understanding, treatment and prognosis of MS (2, 3). MS often starts with a relapsing-remitting course, characterized by new or aggravated existing neurologic dysfunctions that are followed by episodes of recovery (4, 5). Even though there is no evidence for long-term benefits, intravenous (IV) therapy with high-dose methylprednisolone is recommended as the standard treatment of every acute relapse in Germany (6–8). A short-term benefit is that high-dose corticosteroid therapy can speed up symptomatic recovery after a relapse (9). However, side effects and potentially harmful adverse events are not uncommon (8, 10, 11). Given the lack of high-quality scientific evidence concerning the long-term effectiveness of corticosteroid therapy in relapse management and the potentially harmful side effects, effective interventions are needed that involve patients in the relapse-treatment decision-making process. Relapse treatment is a clinical field where patients should be empowered to make a shared or even autonomous treatment decision (12). Clinical experience and scientific evidence show that people with MS (PwMS) prefer active roles in treatment decisions, but often also feel obliged to commence treatment with corticosteroids in any case (12, 13). This obligation causes uncertainty and distress for many PwMS and contrasts with the lack of high-quality evidence concerning more than short-term effects of corticosteroids in relapse management (12). International guidelines on MS management recommend oral corticosteroids as the first treatment choice as evidence shows that oral therapy with corticosteroids is as effective as IV therapy and less costly (14, 15). Although the recently revised German guideline also reflects this evidence, oral therapy is still not considered as first-line treatment in Germany (7). Here, the cost-intensive inpatient therapy of relapses is a highly prevalent management approach, resulting in direct and indirect costs for each relapse in Germany of 2.955 to 5.249 per patient (16). Meeting patients' information needs, making them aware of their ability to participate in clinical decision-making, and preparing them for a shared decision-making encounter by decision coaching, are key facilitators for shared decision-making in healthcare (17). Interventions based on evidence-based patient information (EBPI) could enable patients to take active roles in the decision-making process and make informed treatment decisions, thus promoting patient-centred care (18). EBPI comprises the provision of evidence-based, understandable, transparent, objective and unbiased health information, thus enabling patients to engage in health-related decision-making (19).

An earlier randomized controlled trial (RCT) with 150 PwMS showed that an EBPI and group training programme on relapse management led to a significant increase in informed decision-making and reduction of IV corticosteroid therapies (20). In the intervention group, participants received an evidence-based information brochure on how to manage relapses, and they participated in a 4-h group training session. Participants in the control group received a standard information leaflet. The groups differed significantly, with 78% of relapses in the intervention group being treated with oral or no corticosteroids, compared to only 56% in the control group (difference 22%, 95% CI 11–31%). Although these findings point to the promise of this educational approach for relapse management, many PwMS are unable to access such structured programmes due to mobility restrictions, travel costs, time constraints, or the absence of local MS services offering this or similar programmes (21). To reduce the burden for PwMS and overcome access barriers, telemedicine has become increasingly popular in recent years, providing specialised care for PwMS (22). Telecommunication tools, including smartphones, laptops and other devices connected to the internet, offer opportunities to practise clinical care from a distance and thereby reduce the access gap for PwMS (23, 24). The current COVID-19 pandemic demonstrated the benefit of internet-based programmes and their potential to improve clinical care.

The POWER@MS2 project aims to leverage this potential by developing an interactive web-based complex intervention to support relapse management for PwMS in Germany. The intervention, termed “ABouts,” is currently being evaluated in a multicentre randomized controlled trial (RCT) with 160 PwMS (registration number on ClinicalTrials.gov: NCT04233970, date of registration: 17.01.2020). Detailed information on this RCT and the accompanying process evaluation is provided in the study protocols (25, 26).

According to the guidance by the UK Medical Research Council (MRC) on developing and evaluating complex interventions, a systematic development process, as well as an assessment of feasibility and piloting, are vital preparatory steps before an intervention can be evaluated and implemented (27). Following the MRC guidance, this paper presents the development, feasibility and pilot testing of the complex intervention ABouts, which aims to support PwMS in relapse treatment decision-making. The feasibility and pilot study reported in this paper was conducted to assess the acceptability of the intervention and test the recruitment procedure for the subsequent RCT.

## Materials and methods

The development, feasibility, and pilot testing of the web-based programme ABouts was guided by the UK MRC framework on developing and evaluating complex interventions and comprises the first two stages of the framework (28).

**Figure 1:**
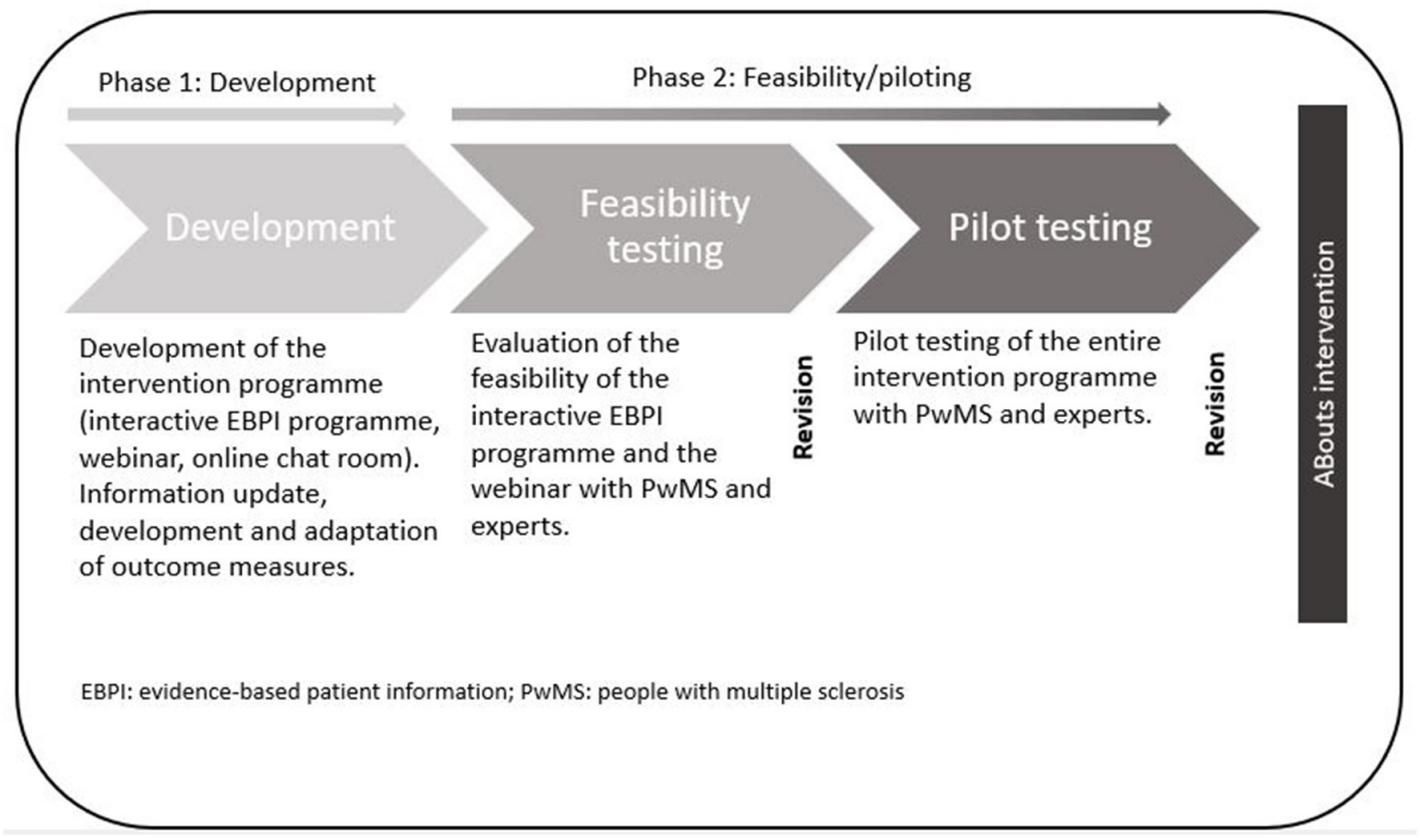
The complex intervention ABouts: development, feasibility, and piloting.

After the development of the intervention and the modelling of outcome measures in the first phase, the feasibility of the intervention components was tested as part of the second phase (28). After revisions, the entire ABouts intervention was tested in a pilot study and revised again. The study flow is illustrated in Figure 1. The Ethical Committee of the University of Lübeck approved the conduct of this research (19–24).

### Development

The first phase included the development of the intervention programme as well as the development and adaptation of outcome measures. The detailed concept of the intervention and outcome measures are outlined in the main study protocol (25). The intervention was designed by a multidisciplinary team (physicians, health scientists, nurses, health economists, statisticians, graphic designers, psychologists and programme developers) (25).

#### Intervention programme

**Figure 2:**
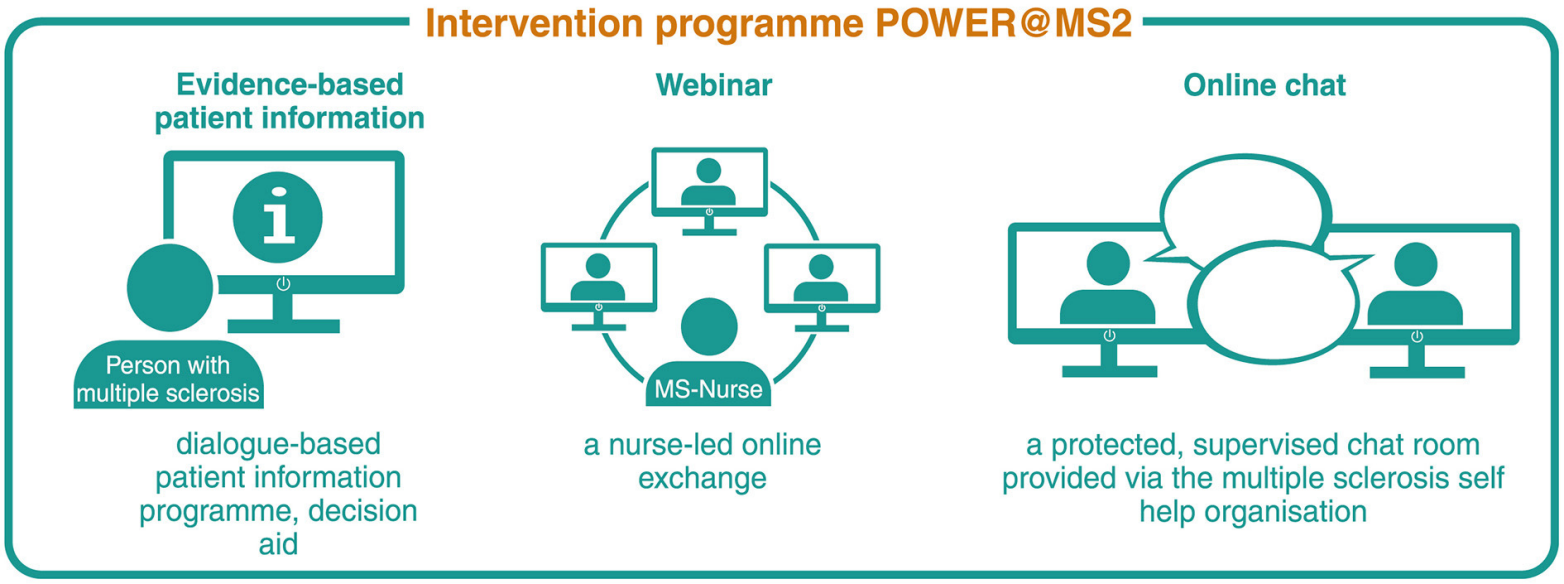
Complex intervention components POWER@MS2. Reproduced with permission from Rahn et al. (29).

The development of the complex intervention programme ABouts, including an interactive, web-based EBPI programme on relapse management, a webinar and an online chat room, started in 2018. All three components, presented in Figure 2, were developed based on the principles of EBPI, the International Patient Decision Aid Standards (IPDAS) and empowerment (30–32). The intervention builds on a patient education programme developed and implemented in previous studies to enhance decision autonomy of PwMS in relapse management (20, 33). An EBPI information brochure that emerged from the patient education programme served as a guideline for the EBPI programme developed in the present study. The information material was transferred, updated (see below) and adapted to a web-based format. The EBPI programme aims to provide participants with evidence-based information on MS and the definition, presentation and evolution of relapses as well as relapse management, mainly focussing on corticosteroid therapy and relapse treatment decision-making (25).

To update the medicine-based information from the brochure, we conducted systematic literature searches addressing the research questions: “What is the effect of corticosteroids in the treatment of relapses in MS?,” “What are the triggering factors of relapses in PwMS?' and ‘What is the prognostic relevance of relapses in MS?.” Between October and December 2018, the database Medline via Ovid was screened. German- and English-language studies were included for further review. Depending on the research question, systematic reviews and/or cohort studies were included. After the exclusion of studies, a full-text screening using a standardised checklist based on the exclusion/inclusion criteria was conducted and the quality of the included papers was critically appraised using a standardised checklist, the Critical Appraisal Skills Programme (CASP) (34). To rate the quality of evidence provided in the programme, we assessed the RCTs included in the systematic reviews on efficacy, side effects and routes of administration of corticosteroids using the Grading of Recommendations Assessment, Development and Evaluation (GRADE) approach (35). This objective and transparent assessment enables relapse treatment decisions to be made based on state-of-the-art research.The initial screening and critical appraisal of the studies were conducted in pairs of two researchers, working independently. Results were compiled and evaluated, and different opinions were discussed until reaching a consensus. Data of all included studies was extracted, cross-checked by an independent researcher, and used to update the information from the brochure. The updated information was transferred to the secure online platform broca^®^, which has already been successfully used for other patient support systems (36, 37).

Development of the platform, transfer and adaptation of the information was performed by GAIA, a public health company that specializes in the development and research of digital therapeutics (38). To ensure comprehensibility of the information, we adapted text to the reading level of secondary school students with the help of the tool “wortliga” (39).

A nurse-led webinar, which constitutes the second component of the complex intervention ABouts, aims to provide a structured online exchange for participants and to clarify questions patients might have (25). To ensure secure communication with participants, we decided to use a web-conferencing tool, which is operated on servers at the coordinating study centre, the MS day clinic in Hamburg.

The third intervention component, an online chat room, was set up as a private, protected group chat on the platform “MS Connect,” operated by the German Multiple Sclerosis Society (DMSG) (40). The chat aims to stimulate a longer-term exchange between participants and offers the possibility to discuss open questions (25).

#### Outcome measures

To assess the primary and secondary endpoints of the main trial (RCT), different questionnaires are used in the course of the study (25). The development phase comprised the adjustment or development of these outcome measures. As part of the process evaluation, which is conducted alongside the RCT, evaluation forms to examine the quality and quantity of implementation of the intervention were developed (26). To measure the health care costs, a health economic questionnaire was developed based on a standardized instrument (41). Standardized questionnaires, such as the Control Preference Scale (CPS) and the Patient Activation Measure (PAM13) were adapted with regard to the focus of the study on relapses and corticosteroid therapy and tested with PwMS (42, 43). New versions of the Planned Behaviour in MS Scale (PBMS) and the Relapse Knowledge questionnaire (RKQ), focusing on relapses and corticosteroid therapy, were developed and also tested with PwMS (44). We evaluated the comprehensibility of all questionnaires, using the think-aloud approach (45). Using the teach-back method, we explored whether the questions were understood correctly or there was a need for clarification (46). The interviews were conducted face-to-face and audio recorded at the coordinating centre. The adapted version of the PBMS questionnaire was then validated in an online survey with 203 PwMS (47). The study also included the adapted version of the RKQ in order to assess knowledge about MS relapses and corticosteroids.

### Feasibility and piloting

#### Feasibility testing

Assessment of feasibility included testing of comprehensibility and acceptability of the EBPI programme and the webinar with PwMS and experts, who are professionally involved in MS (neurologists, a nurse and patient representatives) (25). A convenience sample was recruited via the coordinating study centre by a personal invitation or email. All PwMS aged 18 to 65 years with a relapsing-remitting disease course, or a clinically isolated syndrome and access to the internet were eligible for inclusion. Regarding the study design we did not aim to test for statistical significance and therefore did not pre-determine a specific sample size. After having obtained signed informed consent by all participants, they received a link to the EBPI programme and an evaluation form including closed- and open-ended questions about the programme and the webinar via email. The questions aimed to evaluate the acceptance and relevance of the programme contents and to test the structure and comprehensibility of the information. PwMS were also asked to assess the usefulness of the webinar and the usability of the web-conferencing software. Furthermore, participants were asked to provide demographic and disease-specific data. The degree of disability of participating PwMS was assessed with the Patient Determined Disease Steps (PDDS) questionnaire (48). After this, the webinar was tested with a group of PwMS who were invited via email. The webinar was conducted by one of the researchers (AR) who presented the slides summarising the content of the programme and then led a discussion. Qualitative data was collected by semi-structured telephone interviews with all participants (PwMS and experts). The interviews were based on the results of the evaluation forms, audio-recorded, and notes were taken by the interviewer.

### Pilot testing

Based on the results of the feasibility testing, the intervention was revised and further developed. The subsequent pilot testing was intended to gather information on the usability, accessibility and acceptance of the EBPI programme, the webinar and the chat (25). PwMS were contacted for the pilot testing by email, accessed from an existing email distribution list of the coordinating study centre or through word of mouth by members of the federal association of the DMSG, using a snowballing sampling approach. As with feasibility testing, only PwMS with a clinically isolated syndrome or definite RRMS and access to the internet were eligible to participate. PwMS from the feasibility testing were excluded from participating in the pilot testing. Furthermore, we included experts (patient representatives and a neurologist) to review the EBPI programme, two of whom had already participated in the feasibility testing. Concerning diversity among PwMS we tried to assemble a heterogenous sample with regard to disease course and educational background, by screening eligible participants' demographic data. Signed informed consent was obtained from all participants. The link to the EBPI programme and an evaluation form, which had a similar structure to the one used in the feasibility testing, was sent to all participants by email. The evaluation form focused on questions about the relevance, comprehensibility, and balance of the programme contents. The two experts, who had already participated in the feasibility testing, were also asked about perceived improvements of the programme. After 4 weeks, the webinar was conducted with the group of PwMS, which was moderated by two MS nurses from the coordinating study centre. Following the webinar, PwMS were invited to register and test the private group chat on MS Connect (40). PwMS were also asked to evaluate the webinar and the chat. As with the feasibility testing, qualitative semi-structured telephone interviews were conducted and audio-recorded with all participants. Based on the results of the pilot testing, the intervention was revised and finalised.

### Data analysis

Data from the feasibility and pilot testing as well as the testing of the outcome measures was analysed separately. The quantitative data extracted from the evaluation forms was analysed descriptively using IBM SPSS Statistics version 26.0 and Microsoft Excel (Microsoft Office 365). Instead of using thematic analysis, we clustered the feedback from the audio-recordings of the interviews and the notes taken during the interviews according to themes. This allowed us to provide prompt feedback to the platform developers. All results and necessary changes were discussed and agreed upon in advance by the researchers AR, CH, SK and LW. The results guided the revisions in an iterative process.

## Results

### Development

#### Intervention programme

**Figure 3:**
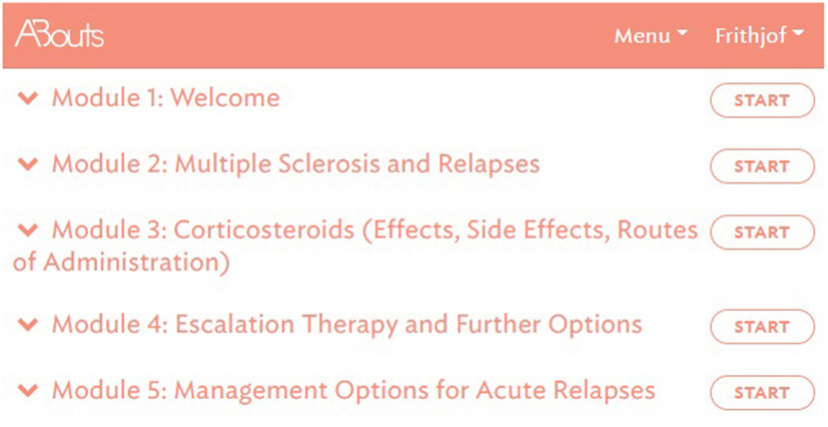
EBPI programme – overview of all modules, © GAIA AG.

Concerning the update of the EBPI programme and the systematic literature searches conducted, a variety of systematic reviews and cohort studies were identified and included. The initial search with regard to the research question “What is the effect of corticosteroids in the treatment of relapses in MS?” retrieved 1,671 citations. Of these, eight systematic reviews were included in the update of the programme. The search concerning the research question “What are the triggering factors of relapses in PwMS?” resulted in 707 citations for systematic reviews and 2,484 for cohort studies, of which three systematic reviews and 14 cohort studies were included. Applying the research question “What is the prognostic relevance of relapses in MS?” resulted in 1,085 citations for systematic reviews and 1,592 for cohort studies, of which two systematic reviews and six cohort studies were included (Supplementary material 1). After data extraction, texts were created or existing texts were adapted and simplified and transferred to the EBPI programme. The final web-based EBPI programme was structured into five modules (see Figure 3, translated into English), comprising the updated information on defining and diagnosing relapses (including differentiation from fluctuations and pseudoexacerbations) and relapse management.

Based on the GRADE rating tool, all RCTs included in module three were categorized using a four-point scale, indicating the certainty of evidence from very low to high (35). The rating included RCTs investigating efficacy and side effects of corticosteroids compared to placebo, efficacy and side effects of oral corticosteroids compared to IV therapy, and RCTs assessing the efficacy of corticosteroids compared to placebo in optic neuritis.

**Figure 4:**
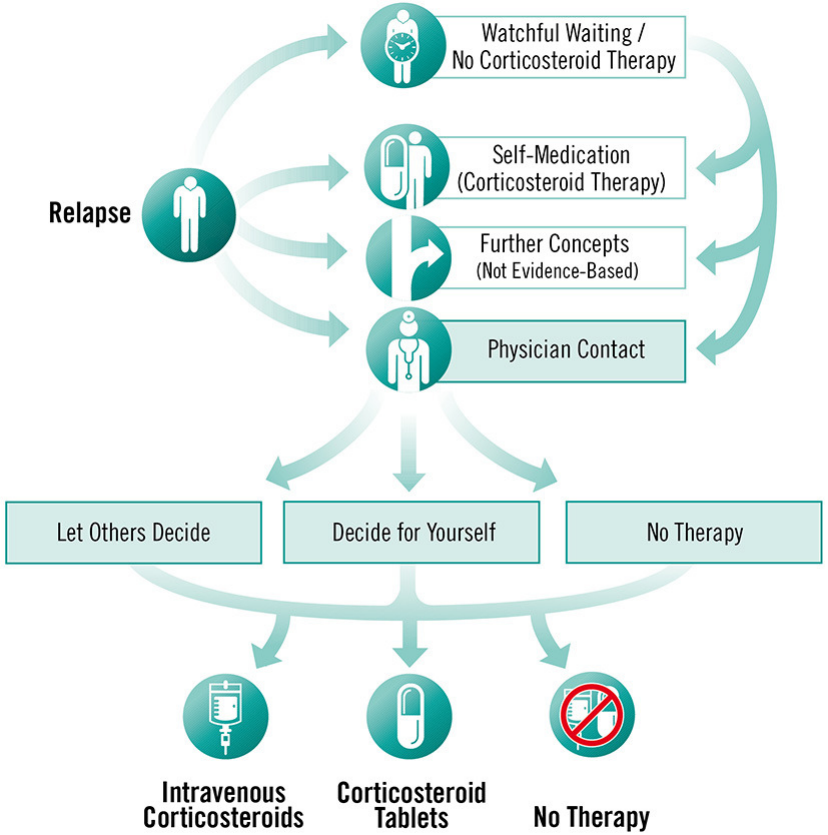
Decision aid for an acute relapse, © Fuchs-Design.

To enable individual processing of modules, the programme is dialogue-based and provides PwMS with tailored information depending on the level of knowledge and interest of the participants (25). Modules are sequentially activated and can be completed only once. However, participants have access to summaries, audio recordings and videos at any time, as well as to a decision aid for an acute relapse as an additional module. The module offers information about all possible actions relevant to the management of an acute relapse and aims to support the decision-making process by empowering PwMS with information about the different options (see Figure 4). In addition, to facilitate decision-making, participants are provided with the link to a decision aid by the German Institute for Quality and Efficiency in Health Care for people facing difficult decisions concerning their health or social life (49).

**Figure 5:**
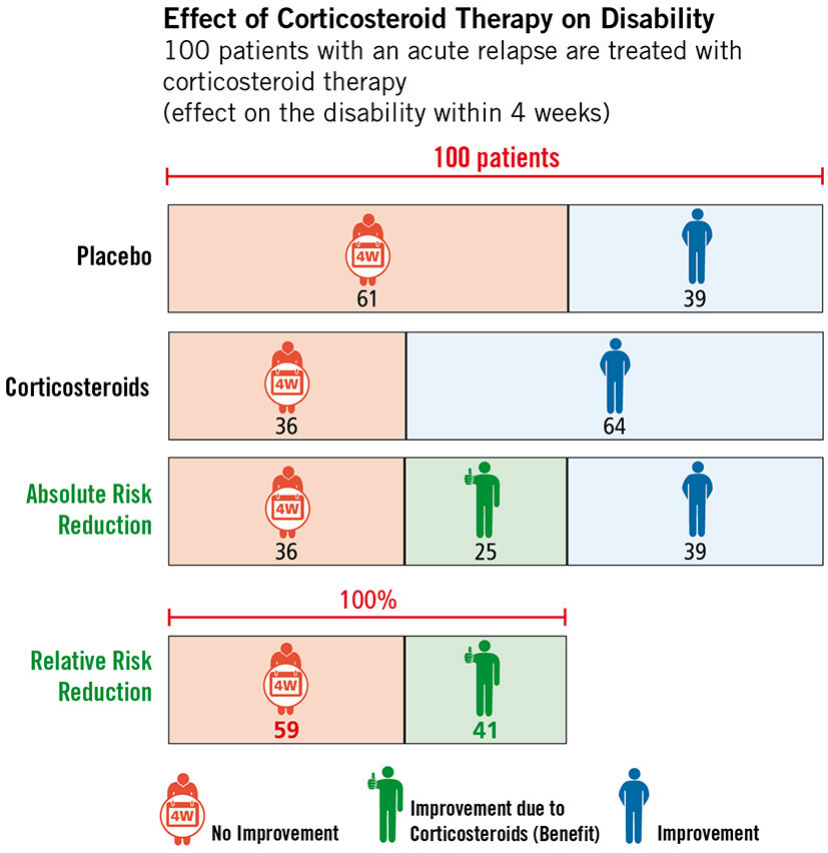
Effect of high-dose corticosteroid therapy on relapse-related disability, © Fuchs-Design.

**Figure 6:**
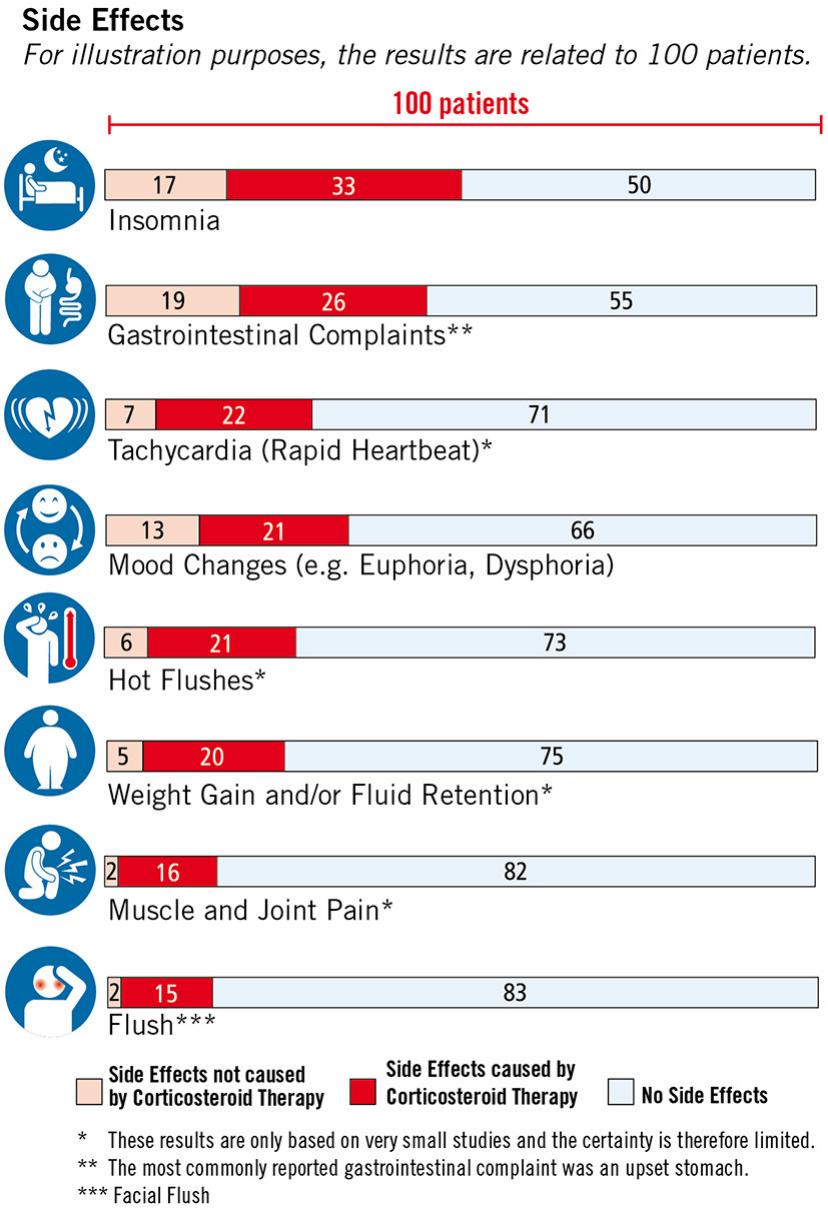
Side effects of corticosteroid therapy, © Fuchs-Design.

The respective studies building the basis of the EBPI programme are available as brief abstracts written in lay language, which can be displayed on request by users. In addition, participants have the opportunity to obtain additional information through the provision of various web links. Module three, which covers the topic of corticosteroid therapy, is the central element of the programme. The literature update and critical appraisal of studies dealing with corticosteroid therapy resulted in the development and embedding of two figures illustrating the effects (see Figure 5) and side effects (see Figure 6) of corticosteroids in the treatment of relapses. Furthermore, module three also includes an explanatory video illustrating the difference between absolute and relative risk reduction.

Due to the sequential activation of the modules, the EBPI programme takes about 4 to 5 weeks to complete (active usage at least once a week for 1 to 1.5 h), including a knowledge quiz on corticosteroid therapy, which is embedded after module five (25). After successful completion of the EBPI programme, the second component of the complex intervention ABouts, the webinar, is conducted with approximately five to ten PwMS. Participants receive the invitation to the webinar and instructions for the web conferencing tool Cisco Webex by email. To accompany the webinar, we developed slides summarising programme content and motivating participants to engage in discussion. The webinar takes ~60–75 min, after which participants are asked to register for the third component of ABouts, the online chat room. The chat is supervised by a patient representative and the study team, who respond to questions and regularly provide input for discussions. The duration of the entire intervention covers a period of a minimum of 5 to 6 weeks with an overall active time commitment of at least 9 h (excluding the chat, which is available to participants for the entire duration of the study).

#### Outcome measures

Testing of outcome measures, which had to be adapted to the present study, confirmed feasibility and comprehensibility. Two questionnaires, the RKQ (manuscript in preparation) and the adapted version of the PBMS, required minimal adjustments. The validation study of the PBMS indicated the construct validity of the instrument and demonstrated that the tool is useful for explaining and predicting relapse treatment decision-making in PwMS (47). Concerning the process evaluation forms, we developed several new forms aimed at different target groups of the study (e.g., PwMS, treating neurologists/study nurses, stakeholders), who are surveyed at different time points in the course of the trial (26).

### Feasibility and piloting

#### Feasibility testing

Feasibility testing was conducted between July and August 2019. Fifteen PwMS with a relapsing-remitting disease course and ten MS experts (patient representatives and neurologists) were selected, of whom five PwMS either declined to take part in the study or did not respond to the invitation email. In total, ten PwMS and ten experts were included in the study. PwMS included seven women and three men with a median age of 51 years (range 24–62). Although we initially planned to include only participants with a clinically isolated syndrome (CIS) or definite RRMS, one participant reported having secondary progressive MS (SPMS). The participants had a median disease duration of 16 years (range 5–37) and all PwMS stated that they had a history of high-dose corticosteroid therapy. Concerning the degree of disability, assessed with the PDDS questionnaire, the participants indicated minor to moderate impairment with a median of 2 (range 0–3) (48).

The group of experts participating in the testing comprised four patient representatives, one nurse and five neurologists. Two neurologists worked in a private practice and three worked at a university hospital. All experts stated that they were either experts or very competent in the field of MS. The detailed characteristics of all participants are summarised in Table 1.

**Table 1:** Participant characteristics.

**Characteristics**	**Feasibility testing**		**Pilot testing**	
	**PwMS**	**Experts**	**PwMS**	**Experts**
	***n* = 10**	***n* = 10**	***n* = 7**	***n* = 3**
**Gender**				
Male	3	4	2	3
Female	7	6	5	0
Age, median (range)	50.5 (24–62)	53 (38–72)	37 (28–57)	54 (40–56)
**Education (highest degree)**				
Secondary school	8	-	7	-
Academic degree	2	-	0	-
**Disease course**				
CIS	0	-	1	-
RRMS	9	-	6	-
SPMS	1	-	0	-
Disease duration (in years), median (range)	16 (5–37)	-	6 (0–33)	-
Duration since last relapse (in years), median (range)	4^*^ (0–22)	-	0 (0–1)	-
PDDS, median (range)	2^*^ (0–3)	-	2 (0–3)	-
Level of expertise in MS				
Excellent (MS expert)	-	5	-	1
Proficient	-	5	-	2
**Professional background**				
Patient representative	-	4	-	2
Nurse	-	1	-	0
Neurologist	-	5	-	1

PwMS, people with multiple sclerosis; CIS, clinically isolated syndrome; RRMS, relapsing remitting multiple sclerosis; SPMS, secondary progressive multiple sclerosis; PDDS, patient determined disease steps.

* missing data for one participant.

**Figure 7:**
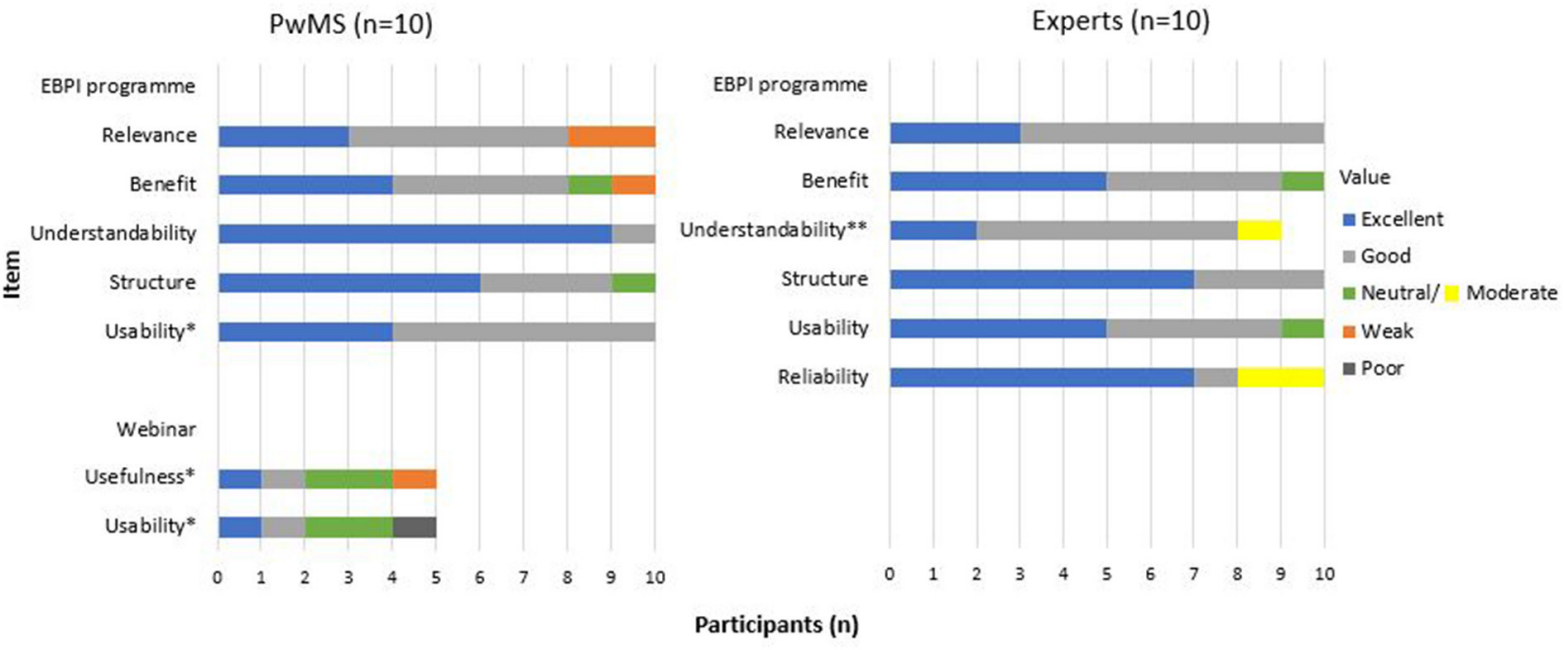
Evaluation questions - feasibility testing.

All PwMS and experts stated that they had reviewed all five modules and the decision aid for an acute relapse. For the overall quality of the programme, PwMS gave a mean rating of 2.33 (*n* = 9), and experts of 1.8 (*n* = 10) (1 = very good – 6 = unsatisfactory). PwMS expressed that they were generally very satisfied with the programme and thought that the programme provided a good orientation on different relapse treatment options. However, some PwMS considered the programme to be better suitable for people with early MS and reported a lack of information on treatment options other than corticosteroids (e.g., vitamin D or practical exercises). Furthermore, PwMS stated that the programme, especially module 3, was partly very text-heavy, and they wished for more key summary points and less repetition. Several PwMS and experts stated that they would prefer more self-determined processing of the individual modules rather than predetermined time intervals after which the individual modules are activated. However, the experts' feedback primarily requested a more balanced presentation of different treatment options for an acute relapse, with the comment, particularly from the neurologists, that other treatment options (such as symptomatic treatments) are not equivalent options to corticosteroids. Also, the experts raised the concern that the neurologists' role in relapse treatment decision-making was not adequately addressed. Concerning the understandability of the EBPI programme, some experts suggested simplifying the texts to be understandable for all levels of education. The detailed results of the feasibility testing are presented in Figure 7. Examples of quotes from PwMS and experts are provided (Supplementary material 2).

##### Revisions

The feedback led to the following revisions in the EBPI programme: (1) The programme settings were changed so that modules one and two were activated at the same time and could be processed consecutively if desired. (2) Practical examples of how to deal with stress were added to the chapter on stress as a triggering factor of relapses. (3) An optional break was integrated to module three. (4) References to neurologists' roles in relapse treatment decision-making and consultation were added in some sections (e.g., in case of severe relapses). (5) Response options of the dialogue-based programme were revised. (6) The decision aid, presenting the various options for action in case of an acute relapse, was slightly modified. (7) Minor formal changes, simplifications of chapters and text reductions were made.

#### Pilot testing

Between September and December 2019, the entire intervention was pilot-tested. In total, thirteen PwMS were contacted via email. Six PwMS did not respond, did not meet the inclusion criteria or declined to take part due to timing issues. Therefore, the final sample consisted of seven PwMS, two men and five women with a median age of 37 years (range 28–57). Due to the generally positive feedback in the feasibility testing, the sample of PwMS included was sufficient.

Three male experts also took part in the testing, two of whom had already taken part in the feasibility testing. The group included two patient representatives and one neurologist working at a university hospital. For more information on participants' characteristics see Table 1.

Asked to judge the overall quality of the programme, PwMS provided a mean rating of 1.57, and experts of 1.67 (1 = very good – 6 = unsatisfactory). The majority of PwMS (*n* = 6) stated that they would recommend the programme to people with early MS immediately after diagnosis or up to 4 years after diagnosis since most questions arise at the beginning of the disease. Only one participant recommended the programme to PwMS at later stages. One expert each stated that he or she would recommend the programme to people with early MS, to PwMS 1 to 4 years after diagnosis or to PwMS 5 to 9 years after diagnosis.

Overall, the pilot testing confirmed the feasibility of the programme and indicated good acceptance in both groups. Although a few PwMS still requested more information on alternative and complementary treatment options, the majority reported having gained important new information and feeling happy to have had the opportunity to access evidence-based patient information in an interactive and easily accessible way. Concerning the usability of the web conferencing tool, the three PwMS who took part in the web conference reported challenges with login and set-up of the programme. After having participated in the web conference, all three participants were invited to register for the online chat room on the platform MS Connect. However, as no exchange occurred via the chat during the pilot testing, the usefulness and usability of the platform could not be fully evaluated.

**Figure 8:**
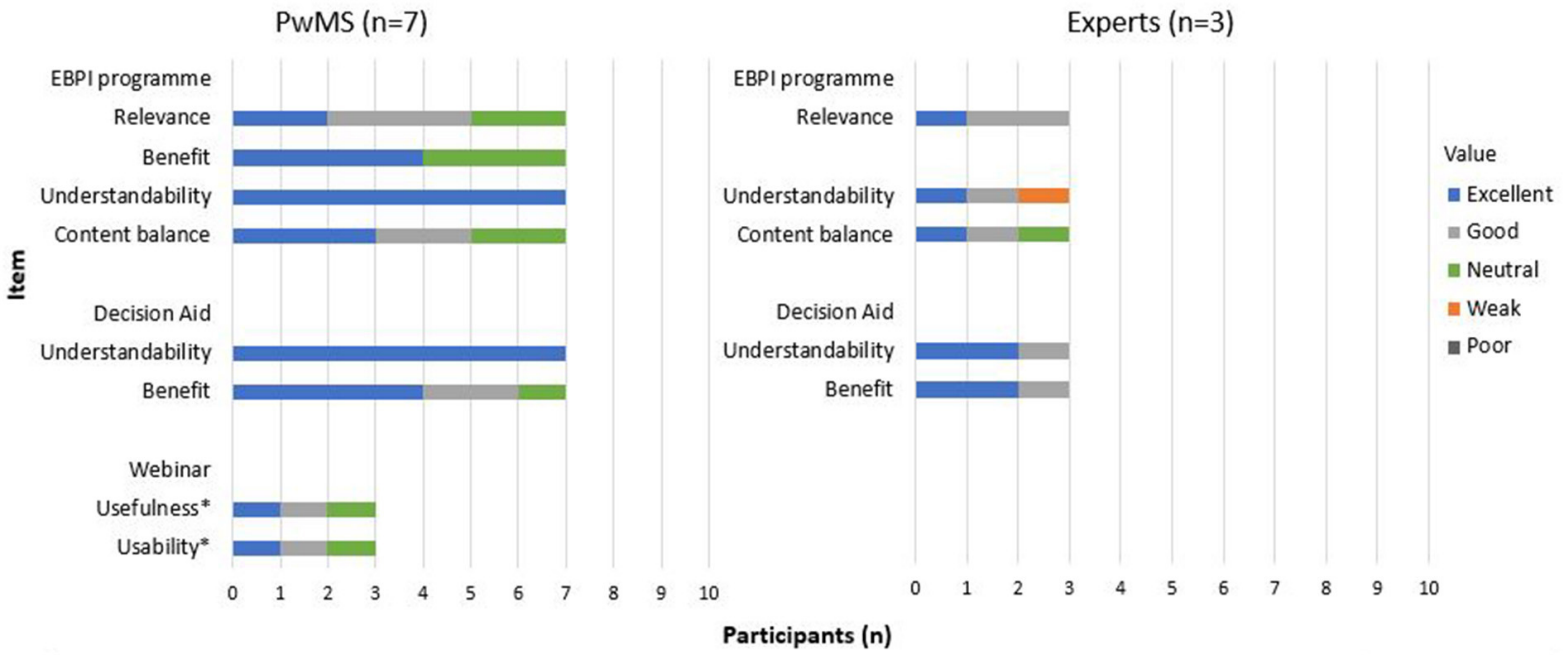
Evaluation questions – pilot testing.

Among the group of experts, the need for revision again focused on a stronger emphasis on the communication between the physician and the patient as well as the neurologists' role in relapse treatment decision-making. One expert mentioned the complexity of the decision aid and commented that the amount of content could cause problems for people with concentration difficulties and fatigue and suggested summarising key points. The two experts who tested the programme for the second time confirmed a relevant improvement of the programme. The detailed results of the pilot testing are presented in Figure 8. Examples of quotes on the individual items are provided (Supplementary material 2).

##### Revisions

Based on the feedback, a final revision was performed, resulting in the following minor modifications of the EBPI programme: (1) Parts of the text were shortened, especially in the third module. Some text passages were replaced by bullet points. (2) In several parts of the texts, more attention was paid to neurologists' roles in relapse treatment decision-making and the consultation. (3) Typographical and spelling errors were corrected.

## Discussion

This paper outlines the development, feasibility testing and piloting of an interactive web-based complex intervention on relapse management for PwMS, following the MRC guidance (27). The results of the feasibility and pilot testing indicate feasibility, user-friendliness and practicability of the programme. An improvement of the programme could be achieved through revisions after feasibility testing. Based on the feedback, we included more PwMS with shorter disease duration in the pilot testing, confirming the feasibility of the programme. The quantitative and qualitative results collected, support our expectations and indicate that the programme is well-accepted by PwMS and experts, including neurologists, patient representatives, and nurses.

Our feasibility and pilot study add to the literature on patient empowerment in relapse-treatment decision-making. Trials show favourable results for telerehabilitation interventions as an alternative method of service delivery for PwMS concerning short-term physical ability and symptoms such as fatigue. However, there is limited evidence to verify what type of intervention is effective (50). Furthermore, only a few studies have investigated decision support tools for PwMS. For example, Kasper et al. showed that providing PwMS with a decision aid on MS immunotherapy led to intensified processing of the information but did not affect the patients' roles in the physician-patient encounter nor the immunotherapy choices made (51). In another study, a 4-h EBPI programme on MS diagnosis, prognosis and immunotherapy was tested with patients with early MS (52). The intervention significantly increased informed choice and risk knowledge and showed trends towards an increased autonomy preference after the intervention. Concerning the acceptability of web-based decision support tools in PwMS, Rahn et al. (53) tested the feasibility of a decision coaching programme on immunotreatment with PwMS, including coaching sessions and access to an online information platform. In line with our findings, the study shows promising results concerning the acceptability and feasibility of the programme and indicates increased informed choices.

Regarding the EBPI programme used for our intervention, Pöttgen et al. tested an interactive online fatigue management programme with PwMS with self-reported fatigue (37). Similar to our EBPI programme, the intervention was based on the artificial intelligence software broca^®^ and resulted in a significant decrease in fatigue.

It was not possible to address the wishes of many PwMS to provide more information on alternative and complementary treatment options in our programme. Although there seems to be a great need, there is hardly any evidence regarding such treatments in the context of relapse management. Thus, the EBPI programme includes an explanation of why these possible treatment options are not equivalent options and thus cannot yet be addressed in an evidence-based tool for PwMS. Furthermore, the experts' wish to emphasise the neurologists' role in relapse treatment decision-making could only partially be addressed, as this would contradict the programme's underlying principle of patient empowerment and autonomous relapse treatment decision-making. However, due to the experts' feedback it was pointed out in several parts of the text, that in case of severe symptoms consultation with the neurologist should take place.

This project demonstrates the benefits of the mixed methods data collection and analysis and the iterative user feedback process in the development of the intervention. The design allows generating insights and an in-depth understanding of the context, impact on different target groups and behavioral barriers to implementing a complex health intervention. A further strength of this study is the systematic, theory-based development process, feasibility testing and piloting of the intervention, involving relevant target groups from different fields of expertise.

Despite these strengths, our study also has some limitations. Due to limited resources and ensuring quick feedback to the platform developers, we clustered the feedback rather than using a systematic, formal analytic approach conducted by independent researchers. However, all results were discussed in team meetings, where different researchers' perspectives were negotiated until reaching a consensus. Furthermore, the study sample was rather small, so that the results cannot easily be generalized. Nevertheless, the sample size was appropriate for the study design and the development of the underlying intervention as this study does not aim to assess clinical impact of the programme. Even though we tried to sample according to educational background, most PwMS who participated in the feasibility and pilot testing had a high level of education. Therefore, we cannot be certain whether PwMS with lower levels of education are also adequately addressed by the intervention. However, we simplified the texts and adapted them to the reading level of secondary school students to make the intervention more understandable for these groups. In addition, the interactive nature of the programme enables the possibility of obtaining in-depth information if needed. Concerning the pilot testing of the online chat room on the platform MS Connect, the usefulness and usability of the platform could not be fully evaluated. Three participants registered for the online chat room, however, no exchange occurred via the chat during the pilot testing.

The programme development, feasibility and pilot testing described in this paper are part of the POWER@MS2 project, which focuses on the development and evaluation of the intervention programme ABouts (25). The intervention builds on the large body of literature supporting patient empowerment and the use of patient-decision aids aiming to increase informed decision-making and improve collaborative communication between physician and patient (1, 18, 32). The patient education programme on MS relapse management, which constitutes the basis for the underlying intervention, was very successful locally and led to more autonomous decision making in PwMS but could not yet be implemented in practice. In accordance with the recently revised German guideline on MS management, which is in line with international guidelines, and recommends shared decision-making and oral corticosteroid relapse treatment, ABouts has the potential to add to a substantial change in relapse management for PwMS in Germany (7). It is expected that the programme will have a positive impact on patients' relapse management and strengthen their autonomy and participation. However, our results show that more research focusing on alternative and complementary treatment options, such as rehabilitation, sports or physiotherapy, are needed for a more comprehensive patient information in MS relapse management.

## Conclusion

We developed a web-based programme on relapse management that is feasible in terms of PwMS' and experts' acceptance of the programme and its applicability in relapse treatment decision-making. The innovative approach may be a low-barrier and cost-effective option to support patients and physicians and facilitate the decision-making process. The intervention is currently evaluated in an RCT with an accompanying cost and process evaluation. If the RCT demonstrates a benefit of the intervention, the intervention could be made accessible to all PwMS in Germany and similar platforms could be developed with focus on, for example, rheumatic diseases or chronic infectious diseases. Furthermore, the intervention could be adapted by other countries.

## Data availability statement

The raw data supporting the conclusions of this article will be made available by the authors, without undue reservation.

## Ethics statement

The studies involving human participants were reviewed and approved by the Ethics Committee of the University of Lübeck, Ratzeburger Allee 160, Building 21, 23562 Lübeck. The patients/participants provided their written informed consent to participate in this study.

## Author contributions

AR, CH, and SK are principal investigators of the POWER@MS2 study and planned the conceptualization, methodology, design, and development of the intervention. LW, AR, CH, and SK discussed and continuously revised the design and planning of the feasibility and pilot study. LW conducted the interviews and performed the data analysis. LW drafted the manuscript with contribution from AR, CH, and SK. JP conducted the systematic literature searches supervised by AR and contributed to the critical appraisal and evaluation of studies as well as data extraction. KG contributed as expert, EF and JS as patient experts in testing and revising the content of the intervention programme. FT and BM contributed to the design and development of the EBPI programme. All authors read, reviewed, and approved the final manuscript.

## Funding

The POWER@MS2 trial, including the development, feasibility and pilot testing of the complex intervention, is funded by the German Innovation Fund of the Federal Joint Committee (GBA), grant number 01VSF17015. The funding body has no influence on the content or the implementation of the study.

## Conflict of interest

Author CH has received research grants, speaker honoraria, and travel grants from Biogen, Celgene, Genzyme, Roche, and Merck. Authors FT and BM are employed at GAIA AG, the company that developed and operates the intervention described in this study. The remaining authors declare that the research was conducted in the absence of any commercial or financial relationships that could be construed as a potential conflict of interest.

## Publisher's note

All claims expressed in this article are solely those of the authors and do not necessarily represent those of their affiliated organizations, or those of the publisher, the editors and the reviewers. Any product that may be evaluated in this article, or claim that may be made by its manufacturer, is not guaranteed or endorsed by the publisher.

## References

[1] World Health Organisation. Atlas Multiple Sclerosis Resources in the World 2008. Geneva: WHO Press (2008). 56 p.

[2] Coetzee T Thompson AJ. Atlas of MS 2020: Informing global policy change. Mult Scler SAGE Publications Ltd. (2020) 3:8811. 10.1177/1352458520968811 33174499

[3] Walton C King R Rechtman L Kaye W Leray E Marrie RA. Rising prevalence of multiple sclerosis worldwide: insights from the Atlas of MS, third edition. Mult Scler. (2020) 3:70841. 10.1177/1352458520970841 33174475 PMC7720355

[4] Mckay KA Kwan V Duggan T Tremlett H. Risk factors associated with the onset of relapsing-remitting and primary progressive multiple sclerosis: a systematic review. Biomed Res Int. (2015) 34:7238. 10.1155/2015/817238 25802867 PMC4329850

[5] Lublin FD Reingold SC Cohen JA Cutter GR Sørensen PS Thompson AJ. Defining the clinical course of multiple sclerosis: the 2013 revisions. Neurology. (2014) 83:278. Available online at: /pmc/articles/PMC4117366/ (accessed February 25, 2022). 24871874 10.1212/WNL.0000000000000560 PMC4117366

[6] Kip M Wiendl H. Therapie der Multiplen Sklerose. Stufentherapie. In: Kip M Schönfelder T Bleß HH, editors. Weißbuch Multiple Sklerose Versorgungssituation in Deutschland. Berlin, Heidelberg: Springer; 2016. p. 56–74. 10.1007/978-3-662-49204-8

[7] Kompetenznetz Multiple Sklerose. S2k-Leitlinie Diagnose und Therapie der Multiplen Sklerose, Neuromyelitis Optica Spektrum und MOG-IgGassoziierte Erkrankungen. (2020). Available online at: https://dgn.org/neuronews/neuronews/konsultationsfassung-der-s2k-leitlinie-diagnose-und-therapie-der-multiplen-sklerose/ (accessed October 4, 2022)

[8] Citterio A la Mantia L Ciucci G Candelise L Brusaferri F Midgard R. Corticosteroids or ACTH for acute exacerbations in multiple sclerosis. Cochrane Database of Syst Rev. (2000) 5:1331. 10.1002/14651858.CD001331 11034713 PMC11391333

[9] Costello J Njue A Lyall M Heyes A Mahler N Philbin M. Efficacy, safety, and quality-of-life of treatments for acute relapses of multiple sclerosis: results from a literature review of randomized controlled trials. Degener Neurol Neuromuscul Dis. (2019) 9:55. 10.2147/DNND.S208815 (accessed June 27, 2022). 31308790 PMC6613013

[10] Gal RL Vedula SS Beck R. Corticosteroids for treating optic neuritis. Cochrane Database of Syst Rev John Wiley and Sons Ltd. (2015) 35:1430. 10.1002/14651858.CD001430.pub4 26273799 PMC4730547

[11] Chrousos GA Kattah JC Beck RW Cleary PA Side Side effects of glucocorticoid treatment: experience of the optic neuritis treatment trial. JAMA. (1993) 269:2110–2. 10.1001/jama.269.16.2110 8468765

[12] Köpke S Heesen C Kasper J Mühlhauser I. Steroid treatment for relapses in multiple sclerosis - The evidence urges shared decision-making. Acta Neurol Scand. (2004) 110:1–5. 10.1111/j.1600-0404.2004.00284.x 15180800

[13] Heesen C Rgen Kasper J Segal J Kö Pke S Mü Hlhauser I. Decisional role preferences, risk knowledge and information interests in patients with multiple sclerosis. Available online at: www.multiplesclerosisjournal.com 15584489 10.1191/1352458504ms1112oa

[14] National Clinical Guideline Centre (UK). Multiple sclerosis: Management of multiple sclerosis in primary and secondary care. London: National Institute for Health and Care Excellence (UK) (2014). Available from: https://www.nice.org.uk/guidance/cg186 (accessed April 30, 2020). 25340249

[15] Burton JM O'Connor PW Hohol M Beyene J. Oral versus intravenous steroids for treatment of relapses in multiple sclerosis. Cochrane Database Syst Rev. (2009) 3:6921. 10.1002/14651858.CD006921.pub2 19588409

[16] Zimmermann A Schönfelder T. Gesundheitsökonomische Aspekte der Versorgung der Multiplen Sklerose. In: Kip M Schönfelder T Bleß HH, editors. Weißbuch Multiple Sklerose: Versorgungssituation in Deutschland. Berlin, Heidelberg: Springer Berlin Heidelberg (2016). p. 95–114. 10.1007/978-3-662-49204-8_5

[17] Joseph-Williams N Edwards A Elwyn G. Power imbalance prevents shared decision making. BMJ. (2014) 348. Available online at: https://www.bmj.com/content/348/bmj.g3178 (accessed May 29, 2020). 10.1136/bmj.g3178 25134115

[18] Köpke S Heesen C Kasper J Mühlhauser I. Steroid treatment for relapses in multiple sclerosis - the evidence urges shared decision-making. Acta Neurol Scand. (2004) 110:1–5. Available online at: http://doi.wiley.com/10.1111/j.1600-0404.2004.00284.x (accessed October 4, 2020). 15180800 10.1111/j.1600-0404.2004.00284.x

[19] Bunge M Mühlhauser I Steckelberg A. What constitutes evidence-based patient information? Overview of discussed criteria. Patient Educ Couns. (2010) 78:316–28. Available online at: https://pubmed.ncbi.nlm.nih.gov/20005067 (accessed February 25, 2020). 20005067 10.1016/j.pec.2009.10.029

[20] Köpke S Kasper J Mühlhauser I Nubling M Heesen C. Patient education program to enhance decision autonomy in multiple sclerosis relapse management: a randomized-controlled trial. Mult Scler. (2009) 15:96–104. 10.1177/1352458508095921 18845657

[21] Marziniak M Brichetto G Feys P Meyding-Lamadé U Vernon K Meuth SG. The use of digital and remote communication technologies as a tool for multiple sclerosis management: narrative review. JMIR Rehabil Assist Technol J Med Int Res. (2018) 4:7805. 10.2196/preprints.7805 29691208 PMC5941090

[22] Block VJ Bove R. Multiple sclerosis minute: telemedicine and multiple sclerosis - practical neurology. Pract Neurol. (2019). Available online at: https://practicalneurology.com/articles/2019-mar-apr/multiple-sclerosis-minute-telemedicine-and-multiple-sclerosis (accessed April 30, 2020).

[23] Ray Dorsey E Topol EJ. State of telehealth. New England Journal of Medicine. Massachussetts Medical Society (2016) 375:154–61. Available online at: http://www.ncbi.nlm.nih.gov/pubmed/27410924 (accessed April 21, 2020). 10.1056/NEJMra1601705 27410924

[24] Yeroushalmi S Maloni H Costello K Wallin MT. Telemedicine and multiple sclerosis: a comprehensive literature review. J Telemed Telecare. (2019) 35:1357633X1984009. Available online at: http://journals.sagepub.com/doi/10.1177/1357633X19840097 (accessed April 21, 2020). 31042118 10.1177/1357633X19840097

[25] Rahn AC Wenzel L Icks A Stahmann A Scheiderbauer Jutta Grentzenberg. Development and evaluation of an interactive web-based decision-making programme on relapse management for people with multiple sclerosis (POWER@MS2) – study protocol for a randomized controlled trial. Trials. (2020) 3:5059. 10.1186/s13063-021-05059-1 33583424 PMC7882468

[26] Wenzel L Heesen C Scheiderbauer J van de Loo M Köpke S Rahn AC. Evaluation of an interactive web-based programme on relapse management for people with multiple sclerosis (POWER@MS2): study protocol for a process evaluation accompanying a randomized controlled trial. BMJ Open. (2021) 11:46874. Available online at: http://bmjopen.bmj.com/ (accessed October 3, 2020). 34598981 10.1136/bmjopen-2020-046874 PMC8488740

[27] Craig P. Developing and evaluating complex interventions. Available online at: www.mrc.ac.uk/complexinterventionsguidance (accessed January 14, 2021).

[28] Craig P Dieppe P Macintyre S Mitchie S Nazareth I Petticrew M. Developing and evaluating complex interventions: the new medical research council guidance. BMJ. (2008) 337:1655. 10.1136/bmj.a1655 18824488 PMC2769032

[29] Rahn AC Wenzel L Icks A Stahmann A Scheiderbauer J Grentzenberg K. Development and evaluation of an interactive web-based decision-making programme on relapse management for people with multiple sclerosis (POWER@MS2)—study protocol for a randomised controlled trial. Trials. (2021) 22:1–15. Available online at: https://trialsjournal.biomedcentral.com/articles/10.1186/s13063-021-05059-1 (accessed August 23, 2020). 33583424 10.1186/s13063-021-05059-1 PMC7882468

[30] Lühnen J Albrecht M Mühlhauser I Steckelberg A. Leitlinie evidenzbasierte Gesundheitsinformation. Evidenzbasierte Leitlinie. (2017). Available online at: https://www.leitlinie-gesundheitsinformation.de (accessed June 23, 2020).

[31] Volk RJ Llewellyn-Thomas H Stacey D Elwyn G. Ten years of the International Patient Decision Aid Standards Collaboration: evolution of the core dimensions for assessing the quality of patient decision aids. (2012). Available online at: http://decisionaid.ohri.ca (accessed April 30, 2020). 10.1186/1472-6947-13-S2-S1 PMC4044280 24624947

[32] Bravo P Edwards A Barr PJ Scholl I Elwyn G McAllister M. Conceptualizing patient empowerment: a mixed methods study. BMC Health Serv Res. (2015) 15:252. Available online at: http://bmchealthservres.biomedcentral.com/articles/10.1186/s12913-015-0907-z (accessed June 23, 2021). 26126998 10.1186/s12913-015-0907-z PMC4488113

[33] Köpke S Richter T Kasper J Mühlhauser I Flachenecker P Heesen C. Implementation of a patient education program on multiple sclerosis relapse management. Patient Educ Couns. (2012) 86:91–7. 10.1016/j.pec.2011.03.013 21507595

[34] Critical Appraisal Skills Programme. CASP Critical Appraisal Skills Programme. CASP Qualitative Checklist. (2018). p. 1. Available online at: https://casp-uk.net/ (accessed January 26, 2021).

[35] Guyatt GH Oxman AD Vist GE Kunz R Falck-Ytter Y Alonso-Coello P. GRADE: An emerging consensus on rating quality of evidence and strength of recommendations. BMJ. (2008) 336:924–6. Available online at: https://www.bmj.com/content/336/7650/924 (accessed January 27, 2021). 18436948 10.1136/bmj.39489.470347.AD PMC2335261

[36] Fischer A Schröder J Vettorazzi E Wolf OT Pöttgen J Lau S. An online programme to reduce depression in patients with multiple sclerosis: a randomised controlled trial. Lancet Psychiatry. (2015) 2:217–23. Available online at: https://pubmed.ncbi.nlm.nih.gov/26359900/ (accessed January 26, 2021). 26359900 10.1016/S2215-0366(14)00049-2

[37] Pöttgen J Moss-Morris R Wendebourg JM Feddersen L Lau S Köpke S. Randomized controlled trial of a self-guided online fatigue intervention in multiple sclerosis. J Neurol Neurosurg Psychiatry. (2018) 89:970–6. Available online at: https://pubmed.ncbi.nlm.nih.gov/29549193/ (accessed January 21, 2021). 29549193 10.1136/jnnp-2017-317463

[38] GAIA- Digital Therapeutics – Science. Available online at: https://gaia-group.com/en/ (accessed January 26, 2021).

[39] Textanalyse-Software, Tipps fürs Schreiben, Text-Seminare – WORTLIGA. Available online at: https://wortliga.de/ (accessed January 27, 2021).

[40] MS Connect. Available online at: https://www.msconnect.de/ (accessed January 27, 2021).

[41] Chernyak N Ernsting C Icks A. Pre-test of questions on health-related resource use and expenditure, using behaviour coding and cognitive interviewing techniques. BMC Health Serv Res. (2012) 12:303. Available online at: https://bmchealthservres.biomedcentral.com/articles/10.1186/1472-6963-12-303 (accessed January 28, 2020). 22950744 10.1186/1472-6963-12-303 PMC3470966

[42] Solari A Giordano A Kasper J Drulovic J van Nunen A Vahter L. Role preferences of people with multiple sclerosis: image-revised, computerized self-administered version of the control preference scale. Oreja-Guevara C, editor. PLoS ONE. (2013) 8:e66127. Available online at: https://dx.plos.org/10.1371/journal.pone.0066127 (accessed January 28, 2020). 23823627 10.1371/journal.pone.0066127 PMC3688863

[43] Zill JM Dwinger S Kriston L Rohenkohl A Härter M Dirmaier J. Psychometric evaluation of the German version of the patient activation measure (PAM13). BMC Public Health. (2013) 13:1027. Available online at: http://bmcpublichealth.biomedcentral.com/articles/10.1186/1471-2458-13-1027 (accessed January 30, 2020). 24172020 10.1186/1471-2458-13-1027 PMC4228438

[44] Kasper J Köpke S Fischer K Schäffler N Backhus I Solari A. Applying the theory of planned behaviour to multiple sclerosis patients decisions on disease modifying therapy - Questionnaire concept and validation. BMC Med Inform Decis Mak. (2012) 12:60. Available online at: /pmc/articles/PMC3416666/?report=abstract (accessed January 28, 2020). 22747904 10.1186/1472-6947-12-60 PMC3416666

[45] Buber R. Denke-Laut-Protokolle. In: Qualitative Marktforschung. Gabler (2009). p. 555–68. Available online at: https://link.springer.com/chapter/10.1007/978-3-8349-9441-7_35 (accessed January 30, 2020).

[46] Schillinger D Piette J Grumbach K Wang F Wilson C Daher C. Closing the loop: Physician communication with diabetic patients who have low health literacy. Arch Intern Med. (2003) 163:83–90. Available online at: https://pubmed.ncbi.nlm.nih.gov/12523921/ (accessed January 29, 2021). 12523921 10.1001/archinte.163.1.83

[47] Haker M Heesen C Wenzel L Köpke S Rahn AC Kasper J. Decision-making about corticosteroids in relapses of multiple sclerosis – development of a questionnaire based on the theory of planned behaviour. Mult Scler Relat Disord. (2021) 55:103182. Available online at: https://linkinghub.elsevier.com/retrieve/pii/S2211034821004491 (accessed August 16, 2021). 34358850 10.1016/j.msard.2021.103182

[48] Learmonth YC Motl RW Sandroff BM Pula JH Cadavid D. Validation of patient determined disease steps (PDDS) scale scores in persons with multiple sclerosis. BMC Neurol. (2013) 13. Available online at: http://www.biomedcentral.com/1471-2377/13/37 (accessed February 11, 2021). 10.1186/1471-2377-13-37 PMC3651716 23617555

[49] IQWIG. Zum Ausfüllen: eine Entscheidungshilfe. (2020). Available online at: https://www.gesundheitsinformation.de/zum-ausfuellen-eine-entscheidungshilfe.html (accessed May 26, 2020).

[50] Khan F Amatya B Kesselring J Galea MPG. Telerehabilitation for persons with multiple sclerosis. A Cochrane review. Eur J Phys Rehabil Med. (2015) 51:311–25. 10.1002/14651858.CD010508.pub2 25943744

[51] Kasper J Köpke S Mühlhauser I Nübling M Heesen C. Informed shared decision making about immunotherapy for patients with multiple sclerosis (ISDIMS): a randomized controlled trial. Eur J Neurol. (2008) 15:1345–52. Available online at: https://pubmed.ncbi.nlm.nih.gov/19049552/ (accessed March 11, 2020). 19049552 10.1111/j.1468-1331.2008.02313.x

[52] Köpke S Kern S Ziemssen T Berghoff M Kleiter I Marziniak M. Evidence-based patient information programme in early multiple sclerosis: a randomised controlled trial. J Neurol Neurosurg Psychiatry. (2014) 85:411–8. Available online at: https://pubmed.ncbi.nlm.nih.gov/24104856/ (accessed March 11, 2020). 24104856 10.1136/jnnp-2013-306441

[53] Rahn AC Köpke S Backhus I Kasper J Anger K Untiedt B. Nurse-led immunotreatment DEcision Coaching In people with Multiple Sclerosis (DECIMS) - Feasibility testing, pilot randomized controlled trial and mixed methods process evaluation. Int J Nurs Stud. (2018) 78:26–36. Available online at: http://www.ncbi.nlm.nih.gov/pubmed/28982479 (accessed June 30, 2020). 28982479 10.1016/j.ijnurstu.2017.08.011

