# Supplementary material for: An interactive web-based programme on relapse management for people with multiple sclerosis (POWER@MS2) - development, feasibility, and pilot testing of a complex intervention
Source: Front Neurol. 2022 Sep 23;13:914814. doi: 10.3389/fneur.2022.914814 (PMC9538652; doi:10.3389/fneur.2022.914814)
Supplement: Supplementary file 1 [file Data_Sheet_1.docx]

Supplementary Material


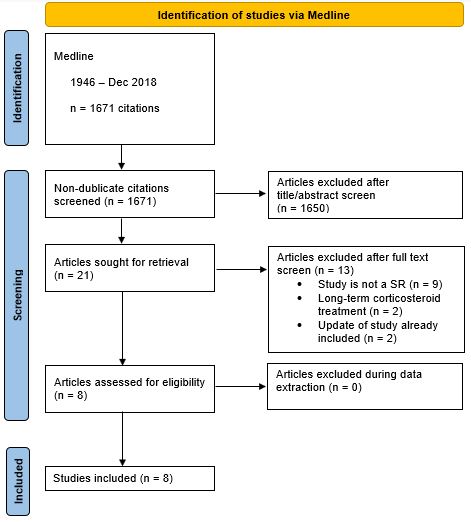


**Supplementary Figure 1.** Flow diagram for systematic reviews(1) – ‘What is the effect of corticosteroids in the treatment of relapses in MS?’


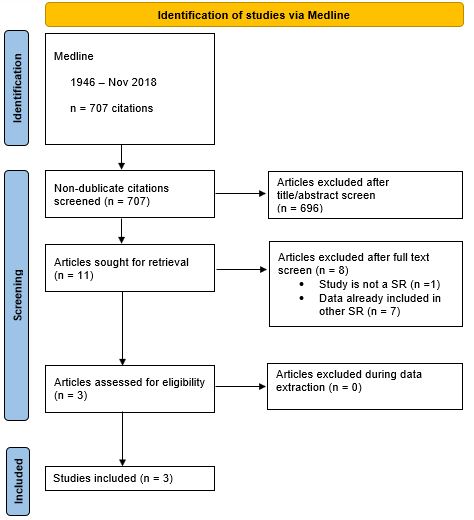


**Supplementary Figure 2.** Flow diagram for systematic reviews(1) – ‘What are the triggering factors of relapses in PwMS?’


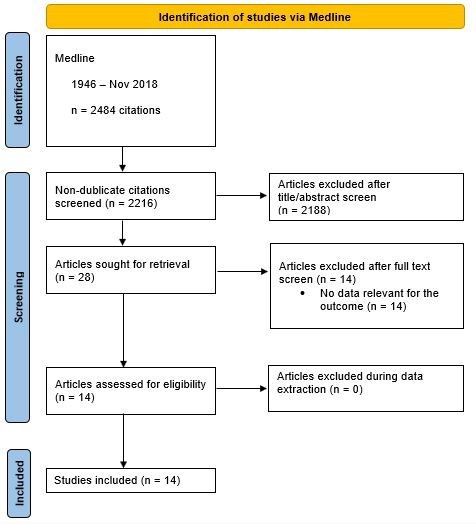


**Supplementary Figure 3.** Flow diagram for cohort studies(1) – ‘What are the triggering factors of relapses in PwMS?’


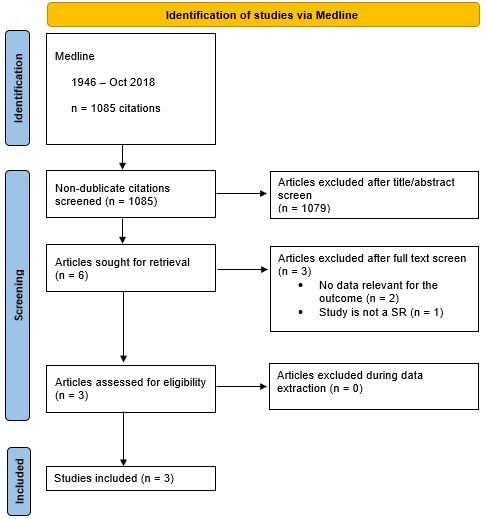


**Supplementary Figure 4.** Flow diagram for systematic reviews(1) – ‘What is the prognostic relevance of relapses in MS?’


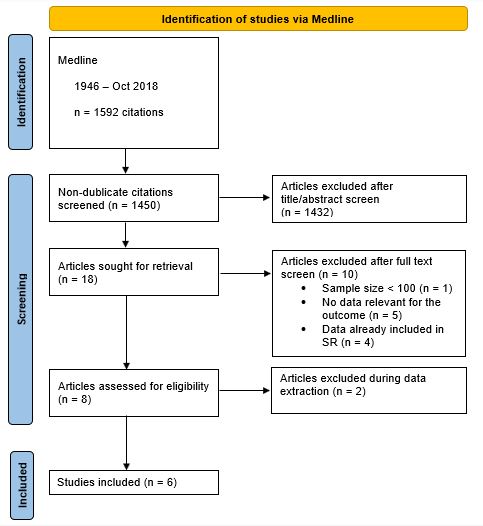


**Supplementary Figure 5.** Flow diagram for cohort studies(1) – ‘What is the prognostic relevance of relapses in MS?’

**References**

1. Page M, McKenzie J, Bossuyt P, Boutron I, Hoffmann T, Mulrow C. The PRISMA 2020 statement: an updated guideline for reporting systematic reviews. BMJ. 2021;372.

**Supplementary Table 1.** PwMS’ and experts’ feedback on the web-based programme on relapse management: qualitative themes and examples of quotes from the feasibility and pilot testing

| **Qualitative Themes** |  | **Exemplary Quotes**  Feasibility testing | | Pilot testing | | |
| --- | --- | --- | --- | --- | --- | --- |
| **EBPI programme** | |  |  |  |  |  |
| Relevance | | “Regarding alternative treatment options, I would have liked more text ... how techniques work. What do I have to pay attention to.” (PwMS) | | „I missed information on alternative treatment options, like traditional Chinese medicine, physiotherapy or occupational therapy.“ (PwMS) | | |
| Benefit | | “The more you know, the more confident you feel and the easier it is to make [relapse treatment] decisions.” (PwMS)  “A prerequisite [for the success of the programme] is a broad discussion about patient self-medication in the Neurological Society, the KKNMS [interdisciplinary MS research network] and the medical advisory board of the DMSG [German Multiple Sclerosis Society].” (Expert) | | “This is a very detailed programme, where you can see that people have thought about how to reduce the fear of the disease in patients by providing information … it is super understandable and there is always the possibility to get more information if needed. Everyone should have access to the programme, I see it as a benefit for all parties [patients and physicians].” (PwMS)  “People have much better access to new research through the programme.” (PwMS) | | |
| Understandability | | “The topics are partly very complicated, but cannot always be simplified, for example the information on absolute risk reduction and relative risk reduction.” (Expert) | | “The language is well chosen, understandable to everyone and clear.” (PwMS)  “For PwMS the content of the programme is very complex, which might lead to problems for people struggling with concentration disorders and fatigue.” (Expert) | | |
| Structure | | “The modules are well structured ... but I would wish that the programme does not limit the number of modules I work on, but that I can decide freely.” (Expert) | |  | | |
| Usability | | “I navigated my way through the programme very well.” (PwMS) | |  | | |
| Reliability | | “The data is correct, and the programme is credible. However, it lacks reference to standards, such as the established standard that relapses are treated with corticosteroids. This missing reference can later lead to conflicts between the patient and the physician.” (Expert) | |  | | |
| Content balance | |  | | “The integration of weblinks and the possibility to obtain more information as an option is great.” (PwMS)  “Even if the [relapse treatment] decision has to be made by the patients themselves, the modules should always point out the relevance of the communication between the physician and the patient.” (Expert) | | |
| **Decision aid** | | | | | | |
| Understandability | |  | | “The decision aid is very complex and should be simplified - maybe only arguments for and against [relapse treatment].” (Expert) | | |
| Benefit | |  | | „It [the decision aid] provides a feeling of safety. It is important that it is available at all times … important things can be forgotten over time.” (PwMS)  “It is certainly helpful to look at the information again during an acute relapse and to reflect on possible options for action.” (PwMS) | | |
| **Webinar** | | | | | | |
| Usefulness | | “The moderator should allow discussions on personal experiences rather only at the end, in between [the presentation] it was rather disruptive.” (PwMS) | |  | | |
| Usability | | “Initially I had problems with the sound, but this was fixed quickly.” (PwMS) | | “I had technical problems in the beginning. That absorbed my attention.” (PwMS) | | |
|  | | | | | | |
